# Incidence and Risk Factors for Patient-related Short-term Cancellation of Elective Arthroscopic Surgery: A Case-matched Study

**DOI:** 10.5435/JAAOSGlobal-D-22-00034

**Published:** 2022-04-05

**Authors:** Konrad I. Gruson, Yungtai Lo, Harrison Volaski, Zachary Sharfman, Priyam Shah

**Affiliations:** From the Department of Orthopaedic Surgery, Montefiore Medical Center, Albert Einstein College of Medicine, Bronx, NY (Dr. Gruson and Dr. Sharfman); Albert Einstein College of Medicine (Dr. Lo and Mr. Shah); and Tel Aviv Sackler School of Medicine, Tel Aviv, Israel (Mr. Volaski).

## Abstract

**Introduction:**

Short-term cancellation of elective ambulatory orthopaedic surgery can result in disruption to the process flow of the operating room, with resultant negative financial implications for the health system. The risk factors for patient-related short-term cancellations within 24 hours of the surgical date have not been well defined.

**Methods:**

A retrospective review of a single orthopaedic surgery electronic internal database was done to identify all cancellations from January 1, 2016, through December 31, 2019, which were made within 24 hours of the surgical date. Inclusion criteria included elective arthroscopic procedures canceled solely for patient-related issues. Any cancellation for surgeon-related or ambulatory center–related reasons was excluded. Demographic patient and surgical data, including insurance type, employment status, previous history of cancellation for the same surgery, socioeconomic status based on the Area Deprivation Index, and surgery type, were tabulated. Each cancellation was matched 1:2 with noncanceled cases based on the anatomic site of the arthroscopy scheduled. Multivariable logistic regression was used to examine associations of patient demographic and medical characteristics with surgical cancellation.

**Results:**

There were 4,715 total arthroscopic procedures done during the study period, of which 126 (2.7%) were canceled within 24 hours of the surgery date. The mean age of the canceled cases was 44.9 ± 16.1 years (range, 14 to 77 years), with 46 females (43%) included. The presence of MRI of the involved joint within 6 months of surgery (adjusted odds ratio [aOR], 0.39, 95% confidence interval [CI], 0.17 to 0.91) and current employment (aOR, 0.56, 95% CI, 0.33-0.94) were independently predictive of noncancellation. Current smokers were more likely to cancel within 24 hours of surgery (aOR, 2.63, 95% CI, 1.4-4.9). Finally, having previously canceled the same surgery was significantly associated with a current surgical cancellation (*P* = 0.004).

**Discussion:**

Identification of the factors associated with short-term patient-related cancellation of elective arthroscopy may serve as the basis for preoperative interventions aimed specifically at those more likely to cancel. In turn, these interventions can minimize preventable cancellations.

Orthopaedic surgery has been consistently identified as one of the most common subspecialties experiencing cancellation of elective procedures.^1,2^ The overall rate of surgical cancellation has been reported to range from 9% to as high as 39%, although much variability exists based on the procedures being done, the healthcare system structure being evaluated, and the timing of the cancellation.^3,4^ Most recently, a retrospective study examining cancellations before and on the day of surgery in an elective orthopaedic sports practice in the United States over a 3-year period reported an overall incidence of nearly 13.4%.^3^ The primary reasons for surgical cancellation have been broadly categorized as patient-related, surgeon-related, and/or hospital-related. Patient-related factors, which include issues related to family or work reasons, have been reported to comprise most of the cancellations and may represent potentially avoidable scenarios.^4,5^ Short-term cancellation of surgery can have a detrimental effect on the operating room workflow efficiency and financial well-being of the healthcare system.^2,6,7^ Furthermore, elective surgery cancellation secondary to surgeon-related or hospital-related reasons can often have a detrimental effect on a patient's emotional, physical, and financial well-being.^8,9^

Despite the patient care issues surrounding surgical cancellation, there remains a dearth of existing literature elucidating the risk factors for short-term surgical cancellation of elective ambulatory orthopaedic procedures in the United States.^3^ The existing data regarding the incidence of and factors associated with day-of-surgery cancellations have been predominately derived from health systems structurally different from those in the United States; have combined the results across multiple subspecialties, including orthopaedics; and have included both inpatient-stay and ambulatory procedures, potentially limiting the generalizability of the reported findings.^4,10^ Regardless of a history of heart failure or advanced renal disease, a history of multiple prior canceled surgeries and surgery scheduled for later in the day were found to be independently associated with 24-hour cancellation among same-day admission and ambulatory elective surgery cases.^2^ Knowledge of potentially modifiable risk factors contributing to cancellation can serve as a basis for preoperative interventions aimed at minimizing this risk.^11^ Petrone et al^3^ found that age older than 46.5 years, non-English speaking, current smoking status, and lack of a history of surgery requiring anesthesia were independently associated with cancellation of elective sports medicine procedures. A recent study has demonstrated an increase in ambulatory surgical center utilization and average Medicare reimbursements from 2012 to 2017.^12^ Therefore, a more complete understanding of the factors driving cancellations, particularly within 24 hours of surgery, can serve to support interventions aimed at eliminating these costly and avoidable situations.

The primary purpose of this study was to determine the incidence of patient-related short-term cancellation of elective arthroscopic surgery and the risk factors predictive of these cancellations. Furthermore, we sought to determine whether surgical type was associated with cancellation risk. We hypothesized that patients with lower socioeconomic status, those with a history of prior cancellations, and those who missed prior outpatient evaluations would likely cancel at a higher rate within 24 hours of surgery.

## Methods

The surgical scheduling data for all elective ambulatory arthroscopic procedures done at a single ambulatory surgical center affiliated with an academic tertiary care medical center, including any cases that were canceled within 24 hours of the scheduled surgery, were obtained from the institution's electronic health records system. The data were collected for January 1, 2016, through December 31, 2019. We elected not to include 2020 to date, given the potential confounding cancellation issues related to the COVID pandemic. The Institutional Review Board (IRB) approval was provided by the Albert Einstein College of Medicine. The IRB approval number was 2021-12871.

The inclusion criteria included any patient who was scheduled to undergo an elective ambulatory arthroscopic procedure involving the shoulder, knee, hip, or elbow. Exclusion criteria included an arthroscopic procedure indicated for an emergent clinical issue (infection and fracture), revision arthroscopic procedures, or cancellations within 24 hours of scheduled surgery incurred because of issues related to medical clearance or hospital-related factors (lack of staff or equipment and lack of operating room availability). Furthermore, we excluded workers' compensation cases, given the difficulty in clearly ascertaining current employment status from the available medical records. Demographic data including patient age, sex, preferred spoken language (English versus non-English), marital status, insurance status (Medicaid versus Medicare versus commercial insurance), smoking history (current versus former versus nonsmoker), employment status, socioeconomic status, type of arthroscopic procedure done (knee versus shoulder versus others), history of prior surgical cancellations for the same procedure, the number of MRIs done within 6 months before the scheduled surgery, and history of any prior orthopaedic surgical procedure requiring regional and/or general anesthesia. A patient's socioeconomic status was determined by the use of the Area Deprivation Index (ADI), an instrument that allows for ranking of neighborhoods by socioeconomic disadvantage at both the state and national levels.^13,14^ The ADI is a validated, multidimensional tool based on multiple factors, including income, education, employment, and housing quality. At the state level, the data are categorized in deciles (1 to 10), with a score of 1 indicating the least disadvantaged and 10 being the most disadvantaged. At the national level, the data are given as percentiles, with the lower values representing the least disadvantaged. For the purpose of statistical analysis, we categorized the state-level ADI as 1 to 5 versus 6 to 10 and the national-level ADI as ≤25th percentile versus >25th percentile.

A short-term cancellation was defined as an elective procedure included on the final operating room schedule generated on the afternoon before the surgical date and then subsequently canceled before or on the day of surgery. An avoidable cancellation is defined as one that could have been prevented by improved patient-physician instructions. At out institution, an attempt is made to contact the patient to remind them of the arrival time and emphasize preoperative instructions, although it is not universally documented whether actual contact was made. Each cancellation underwent a randomly generated 1:2 matching with noncanceled cases based on clinical indication for surgery of the same joint to create the two cohorts. The use of multiple control subjects per case was used to increase the power of this study. A biostatistician was consulted regarding the study design and data analysis.

Differences in demographic and medical characteristics between patients with and without arthroscopy cancellation were compared using the Wilcoxon rank sum test for age and chi square tests for categorical variables. The Fisher exact test was used to compare the association between the history of prior surgical cancellation and current short-term cancellation, given the lack of prior cancellations in the noncancellation cohort. Logistic regression was used to examine associations of demographic and medical characteristics with arthroscopy cancellation. Any variable with a *P* value of <0.25 in the initial analysis was a candidate for multiple logistic regression, although prior surgical cancellation was excluded from the final model. The zero count for prior cancellation among the current noncancellation cohort precluded estimation of an odds ratio (OR). A *P* value of <0.05 was considered statistically significant. All statistical analyses were conducted using SAS version 9.4 (SAS).

## Results

There were 4,715 total arthroscopic procedures done, of which 126 (2.7%) were canceled within 24 hours of the day of surgery. Of the total cases, there were 1,824 shoulder arthroscopies (39%), 2,647 knee arthroscopies (56%), and 244 hip/elbow arthroscopies (5%) included. Of the cases that were canceled, 106 (84%) were determined to be predominately patient-related and were matched to 212 randomly selected noncanceled cases. The most common reason for patient-related cancellation was that the patient did not adhere to the nothing by mouth (NPO) instructions (30%) (Table 1). The mean age of the cancellation cohort was 44.9 ± 16.1 years (range, 14 to 77 years), with 46 females (43%). The number of missed office visits was not significantly different between the patients who canceled (median 0, interquartile range [IQR] 1; range, 0 to 4) and those who did not (median 0, IQR 0; range 0 to 6) (*P* = 0.15). The number of MRIs of the affected joint obtained within 6 months of surgery was not significantly different between the patients who canceled (median 1, IQR 0; range, 0 to 2) and those who did not (median 1, IQR 0; range 0 to 2) (*P* = 0.35). Patients with Medicare/Medicaid versus commercial insurance (ꭓ^2^ = 6.1, *P* = 0.047), those without active employment (ꭓ^2^ = 7.1, *P* = 0.008), current smokers (ꭓ^2^ = 10.0, *P* = 0.007), those with a history of a prior surgical cancellation (*P* = 0.004), and those without imaging of the affected joint within 6 months of surgery (ꭓ^2^ = 4.3, *P* = 0.039) were more likely to cancel surgery within 24 hours of the surgical date after univariate analysis. Neither patient socioeconomic status as measured by local or national ADI nor preference for a non-English language was significantly associated with an increased risk for cancellation (Table 2). After logistic regression analysis, being employed (adjusted OR [aOR], 0.56, 95% confidence interval [CI], 0.33 to 0.94, *P* = 0.027) and having undergone imaging of the affected joint within the past 6 months (aOR, 0.39, 95% CI, 0.17-0.91, *P* = 0.029) were associated with a significantly lower risk for short-term cancellation. Current smokers were more likely to cancel compared with nonsmokers (aOR, 2.63, 95% CI, 1.42-4.90, *P* = 0.002) (Table 3).

**Table 1:** Patient-related Causes for Short-term Cancellation of Surgery

Variable	N (%)
Patient not NPO	32 (30.2)
Patient illness	23 (21.7)
Patient no-show	22 (20.8)
Procedure rescheduled	16 (15.1)
No escort available	7 (6.6)
Improvement in condition	6 (5.6)
	106 (100)

NPO = nothing by mouth

**Table 2:** Demographics for Short-term Elective Surgical Cancellations and Noncancellations

Variable	All Subjects (n = 318)	No Cancellation (n = 212)	Cancellation (n = 106)	*P* Value
Age, median (IQR)	48 (31-56)	47 (30-56)	48 (32-55)	0.657
Sex, n (%)				0.132
Female	161 (50.6)	101 (47.6)	60 (56.6)	
Male	157 (49.4)	111 (52.4)	46 (43.4)	
Preferred language, n (%)				0.696
English	272 (88.3)	180 (87.8)	92 (89.3)	
Non-English	36 (11.7)	25 (12.2)	11 (10.7)	
Insurance type, n (%)				**0.047**
Medicaid	128 (40.3)	77 (36.3)	51 (48.1)	
Medicare	42 (13.2)	26 (12.3)	16 (15.1)	
Commercial	148 (46.5)	109 (51.4)	39 (36.8)	
Employment status, n (%)				**0.008**
No	194 (61.4)	118 (56.2)	76 (71.7)	
Yes	122 (38.6)	92 (43.8)	30 (28.3)	
ADI, national (%)				0.222
≤25	280 (88.1)	190 (89.6)	90 (84.9)	
>25	38 (11.9)	22 (10.4)	16 (15.1)	
ADI, local decile, n (%)				0.300
1-5	73 (23.0)	45 (21.2)	28 (26.4)	
6-10	245 (77.0)	167 (78.8)	78 (73.6)	
Marriage status, n (%)				0.281
No	234 (73.6)	152 (71.7)	82 (77.4)	
Yes	84 (26.4)	60 (28.3)	24 (22.6)	
Diabetes, n (%)				0.081
No	260 (81.8)	179 (84.4)	81 (76.4)	
Yes	58 (18.4)	33 (15.6)	25 (23.6)	
Cardiac disease, n (%)				0.263
No	179 (56.3)	124 (58.5)	55 (51.9)	
Yes	139 (43.7)	88 (41.5)	51 (48.1)	
Pulmonary disease, n (%)				1.0
No	304 (95.6)	203 (95.7)	101 (95.3)	
Yes	14 (4.4)	9 (4.3)	5 (4.7)	
Renal disease, n (%)				0.066
No	299 (94.0)	203 (95.7)	96 (90.6)	
Yes	19 (6.0)	9 (4.3)	10 (9.4)	
Smoking status, n (%)				**0.007**
Never	186 (58.5)	132 (62.3)	54 (50.9)	
Former	73 (23.0)	51 (24.0)	22 (20.8)	
Current	59 (18.5)	29 (13.7)	30 (28.3)	
History of surgery, n (%)				0.665
No	223 (70.1)	147 (69.3)	76 (71.7)	
Yes	95 (29.9)	65 (30.7)	30 (28.3)	
MRI within 6 mo, n (%)				**0.039**
No	25 (7.9)	12 (5.7)	13 (12.3)	
Yes	293 (92.1)	200 (94.3)	93 (87.7)	
Missed office visit within 6 mo, n (%)				0.063
No	242 (76.1)	168 (79.3)	74 (69.8)	
Yes	76 (23.9)	44 (20.7)	32 (30.2)	
Missed office visit within 6 mo, n (%)				0.151
<2	297 (93.4)	201 (94.8)	96 (90.6)	
≥2	21 (6.6)	11 (5.2)	10 (9.4)	
Prior surgery cancellation, n (%)				**0.004**
No	313 (98.4)	212 (100)	101 (95.3)	
Yes	5 (1.6)	0 (0)	5 (4.7)	

ADI = Area Deprivation Index, IQR = interquartile range. The values were bolded to signify that the p<0.05.

**Table 3:** Factors Associated With Arthroscopy Cancellation on Multivariable Logistic Regression

Characteristic	aOR (95% CI)	*P* Value
MRI within 6 mo		
Yes	0.39 (0.17-0.91)	0.029
No (reference)	1	
Smoking status		
Current	2.63 (1.42-4.90)	0.002
Former	0.96 (0.52-1.77)	0.896
Never (reference)	1	
Employment status		
Yes	0.56 (0.33-0.94)	0.027
No (reference)	1	

aOR = adjusted odds ratio, CI = confidence interval

**Figure 1:**
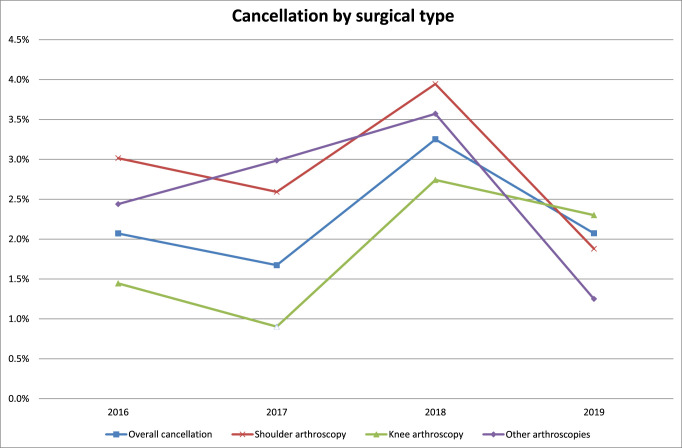
Graph showing annual patient-related cancellation rates stratified by surgical type.

Overall, shoulder arthroscopy cases (3.6%) canceled at a significantly higher rate compared with both knee arthroscopy (2.1%) and other arthroscopies (2.5%) (ꭓ^2^ = 9.2, *P* = 0.009). However, when analyzing only cases canceled for patient-related reasons, there was no significant difference in the cancellation rates based on surgical type (ꭓ^2^ = 4.4, *P* = 0.11) (Figure 1).

## Discussion

The main findings of this study are that those patients without active employment, current cigarette smokers, and those who have not undergone MRI of the involved joint within 6 months of the scheduled procedure were markedly more likely to cancel their surgery within 24 hours of the date of surgery. A history of having canceled the surgery previously was markedly associated with a current cancellation. Approximately 80% of the patient-related cancellations in this study were deemed to be avoidable.

We found that the overall incidence of short-term cancellations for elective arthroscopy over the 4-year period under study was 2.7%, with shoulder arthroscopy procedures experiencing a similar patient-related cancellation rate to both knee and other (hip/elbow) arthroscopies. Furthermore, the patient-related cancellations comprised more than 80% of the total cancellations. The risk of cancellation in our study was lower than that of Petrone et al,^3^ who reported a cancellation rate of 13.4% for primarily elective arthroscopic procedures. This discordance is likely the result of their inclusion of any surgical cancellation before and including the day of surgery. Our short-term cancellation rate was similar to that of Kaddoum et al^10^ who reported a 4.4% cancellation rate of which 71.6% were considered avoidable, although this included elective surgical procedures in multiple subspecialties, including orthopaedic surgery. Laisi et al^15^ reported a cancellation rate of 5.4% for elective orthopaedic procedures, with 72% being patient-related. Furthermore, Caesar et al^4^ reported that 17% of the patients undergoing elective orthopaedic surgery self-canceled, similar to our finding of an approximately 15% self-cancellation rate. By contrast, however, our cancellation for issues related to not being NPO was higher than that of Caesar et al, who reported a rate of 12% lacking sufficient medical clearance and/or preoperative fasting. We did not correlate cancellations related to being NPO to whether the case was scheduled for the morning or the afternoon, although a previous study reported that afternoon-scheduled cases were markedly more likely to cancel than morning cases.^2^ Finally, our finding that shoulder arthroscopy canceled at a similar rate to other anatomic sites is consistent with that of Petrone et al, who reported no notable difference in cancellation between patients undergoing distal biceps repair, in addition to knee, hip, and shoulder arthroscopies. This finding suggests that interventions aimed at reducing short-term cancellation can focus on patient demographics as opposed to the surgical procedure being done.

We found that current smokers had a nearly threefold increased risk for canceling their arthroscopic procedure within 24 hours of surgery when compared with both former smokers and nonsmokers. Interestingly, former smokers were not more likely to cancel surgery as compared with nonsmokers. Our results are consistent with those of Petrone et al,^3^ who reported an OR of 2.3 for short-term cancellation among current smokers compared with nonsmokers, although subgroup analysis of former smokers was not conducted. The association between cigarette smoking and both anxiety and depression has previously been shown,^16,17^ with current smokers having a markedly higher risk for anxiety compared with both former smokers and nonsmokers.^17^ Preoperative anxiety has been reported to be as high as 47% among patients undergoing elective surgery and has been postulated as a possible reason for surgical cancellation.^18^ In this study, a history of prior surgical cancellation for the same surgery was markedly associated with a current cancellation, although an OR could not be estimated, given the lack of prior cancellations in the current noncancellation cohort. Similarly, Tan et al^2^ reported that patients with ≥4 cancellations within the previous 3 years were more likely to cancel. These data can easily be obtained from the patient's medical records, and these patients may warrant more frequent preoperative contact by the physician's office to ensure appearance on the surgical date.

Obtaining MRI of the involved surgical site within 6 months before surgery was found to reduce the risk for same-day cancellation by 61%. Lack of recent imaging may indicate that a procedure has been booked too far in advance and could result in improvement in the patient's symptoms, resulting in cancellation. In this study, we found that 6% of our cancellations resulted from an improvement in clinical symptoms. Yoo et al^19^ reported that improvement in symptoms and fear of economic burden were the most common reasons that patients elected not to undergo arthroscopic rotator cuff surgery for symptomatic full-thickness tears after having been indicated for surgery. Similar conclusions regarding increased cancellation and improved symptoms were found in the setting of carpal tunnel surgery.^20^ Of interest, we found a trend toward an increased cancellation risk with missed outpatient surgical visits within 6 months of surgery, although this association did not hold after multivariable analysis. A previous study reported a markedly higher rate of cancellation (13% versus 9%) among patients who missed ≥4 clinic visits in the year before surgery, a number seen uncommonly among our patients. Finally, we found that currently employed patients were less likely to cancel surgery. Caesar et al^4^ reported that work-related and family-related considerations resulted in 16% of the patient-related orthopaedic cancellations. Employed patients undergoing elective orthopaedic surgery have presumably arranged their work considerations to minimize the effect of the recovery time and thus are more likely to keep the surgical date they have selected.

After multivariable analysis, we found that insurance type was not markedly associated with same-day cancellation. Petrone et al^3^ similarly found no difference in cancellation rates between patients with Medicare/Medicaid versus private insurance after multivariable regression analysis. Furthermore, patient socioeconomic status, as measured by the ADI at both the state and national levels, was also not found to be associated with an increased risk for surgical cancellation. In a study conducted in Singapore which was based on both private and public insurance programs similar to the United States, Tan et al^2^ reported no notable difference in 24-hour cancellation between patients of lower socioeconomic status as defined by whether they were eligible for a subsidized ward. In the same study, however, the authors reported a higher cancellation rate for patients who received financial assistance from MediFund, a national financial assistance program. We found no notable difference in the cancellation risk among patients who preferred conversing in English as compared with those who preferred a non-English language. These findings were discordant with the findings of Petrone et al,^3^ who reported a cancellation rate among non-English speaking patients. Our hospital policy includes the use of a professional language service for non-English speaking patients, particularly when discussing surgical intervention and obtaining surgical consent. This intervention has been demonstrated previously to improve the ability to communicate with patients and obtain informed consent.^21^

There are certainly limitations to our study. We did not match the cases by the actual procedure done, rather only by the surgical anatomic site involved. The potential extensiveness and/or duration of the proposed procedure may itself be a reason why patients elect to cancel surgery, and this may have been underexplored in this study. Furthermore, we involved multiple surgeons in this study, which may play a confounding role regarding the preoperative communication with patients regarding preparation and expectations for the surgery. However, this may also increase the generalizability of our results to other institutions. Finally, we did not seek to assess the efficacy of any specific intervention to reduce the cancellation risk based on the identification of the patient risk factors in this study. A strength of our study is the fact that we included only those patients who canceled on the day of surgery, which may be the most challenging clinical scenario because the opportunity to fill the operating room time with another elective case is difficult and would most benefit from preoperative intervention. The inclusion of multiple surgeons in this study increases its generalizability. Finally, we were able to discern the general reasons for why patients canceled on the same day, again providing an understanding of where the intervention efforts should be focused.

## Conclusion

Most of the short-term cancellations in this study were patient-related and potentially avoidable. Current smokers, those without recent MRI of the affected joint, and unemployed patients represent patients more likely to cancel elective arthroscopic surgery within 24 hours of scheduled surgery. A history of prior surgical cancellation was markedly associated with short-term cancellation, although additional study of this parameter is required, given the low incidence in this study. Given the financial implications of the cancellations, in addition to the negative effect on efficient hospital operations, additional research into the effect of specific intervention strategies aimed at those most at risk is indicated.
